# FUNCTIONAL LIMITATIONS AND WELL-BEING THROUGHOUT THE ADULT LIFESPAN: THE ROLE OF PERCEIVED BURDENSOMENESS

**DOI:** 10.1093/geroni/igae098.3501

**Published:** 2024-12-31

**Authors:** Claire Williams, Natalie Dautovich

**Affiliations:** Virginia Commonwealth University, Richmond, Virginia, United States; Virginia Commonwealth University, Richmond, Virginia, United States

## Abstract

Functional limitations represent individuals’ difficulty with completing essential activities of daily living. Though functional limitations have been linked to poorer well-being outcomes, less is known about potential protective factors for well-being in the lived experience of functional limitations. This study aimed to examine the potential moderating effect of perceived burdensomeness on the association between functional limitations and life satisfaction, a common indicator of well-being, across the adult lifespan. The present study used archival data from 696 individuals who participated in the Midlife in the United States Refresher study. Participants’ perceived burdensomeness significantly moderated the association between functional limitation status and life satisfaction (B = 0.18, p =.0486) after controlling for gender identity (B = 0.003, p = 0.9461) and racial identity (B = 0.03, p = 0.0724). The negative association between functional limitations and life satisfaction was stronger for those with a higher degree of perceived burden. Interestingly, age did not emerge as a significant secondary moderator in this model. Therefore, it seems that the buffering effect of a low level of perceived burden may be salient across age groups. The current study provides support for the importance of perceived burdensomeness in the lived experience of functional limitations across the adult lifespan. In the future, clinicians should consider integrating mental health screeners in healthcare settings in order to identify at-risk individuals who are experiencing functional limitations, and potentially consider establishing preventative, education-based interventions concerning sleep in the experience of functional limitations.

